# Sustaining herd immunity against measles: Insights from a serological cohort study in an outbreak-free population

**DOI:** 10.1007/s15010-026-02832-9

**Published:** 2026-05-27

**Authors:** Miracle Amadi, Simopekka Vänskä, Irja Davidkin, Heikki Haario, Tuija Leino, Merit Melin, Kari Auranen, Mia Kontio

**Affiliations:** 1 School of Engineering Sciences, LUT University, Lappeenranta, Finland; 2 Department of Public Health, Finnish Institute for Health and Welfare, Helsinki, Finland; 3 Department of Mathematics and Statistics, University of Turku, Turku, Finland; 4 Department of Clinical Medicine, University of Turku, Turku, Finland

**Keywords:** Measles, Antibody decline, Transmission, Herd immunity, Estimation

## Abstract

**Purpose:**

Measles antibody levels decline after MMR vaccination, raising concerns about individual protection and maintenance of herd immunity. In Finland, MMR vaccinations started in 1982, and only sporadic measles cases have been observed since the late 1980s. This suggests that a high proportion of individuals are unlikely to transmit measles. We examine the sustainability of herd immunity by inferring the antibody threshold sufficient to interrupt measles transmission.

**Methods:**

We utilized measles antibody data collected after the second MMR vaccine dose from 157 individuals in a Finnish MMR-vaccinated cohort at 8 sampling rounds during 1987–2012 (ages 6–31 years), with antibody concentrations measured using the haemagglutination inhibition assay and/or enzyme immunoassay depending on the round. Antibody decline after vaccination was estimated and extrapolated beyond the study age range to predict the proportion exceeding candidate antibody thresholds in the 2022 Finnish population. Threshold sufficiency was evaluated by comparing this proportion with the critical proportion required to sustain the absence of measles epidemics.

**Results:**

The mean follow-up time was over 20 years, with 68% of all potential samples available. After age 13, antibody levels declined slowly or very slowly, with first and second half-lives of 10 and 20, or 22 and 67 years, respectively. No unique threshold was identified. However, the continued absence of measles epidemics suggests a very slow decline and/or a low (< 120 mIU/ml) antibody threshold to interrupt transmission.

**Conclusions:**

Vaccine-induced protection against measles transmission appears long-lasting. For herd immunity, declining vaccine uptake poses a greater risk than waning immunity.

**Supplementary Information:**

The online version contains supplementary material available at 10.1007/s15010-026-02832-9.

## Introduction

Measles is a highly contagious human virus. In the pre-vaccination era, a vast majority of individuals contracted measles, usually during childhood, and subsequently developed immunity that is thought to be lifelong [1, 2]. Accordingly, measles antibody levels remained relatively stable throughout adulthood [3, 4]. Estimates of measles basic reproduction number (R_0_), the average number of secondary infections generated by a typical measles case in a fully susceptible population, have often exceeded 10 [5]. Due to the high R_0,_ achieving herd immunity requires that over 90% of the population (critical proportion of $\:1-1/{\mathrm{R}}_{0}$), i.e., those having immunity through vaccination or prior infection, act as a firewall to interrupt transmission [6]. Here, *herd immunity* refers to population-level protection sufficient to prevent large-scale measles outbreaks.

Due to effective vaccination programs, endemic measles had been eliminated from many European countries by 2015 [7]. However, since 2017, measles resurged in several of these countries, particularly in those with suboptimal vaccination coverage [8, 9]. As the honeymoon period of vaccination wanes, the accumulation of unvaccinated individuals can undermine herd immunity [10]. In addition to insufficient vaccination coverage, the potential loss of immunity among vaccinated individuals may also contribute to this resurgence [11]. Notably, measles cases have been reported even among twice vaccinated individuals [12, 13].

In Finland, measles vaccination began with a mono-component vaccine in 1975 and changed to a two-dose schedule (at the ages of 12–18 months and 6 years) with the three-component MMR (measles-mumps-rubella) vaccine in 1982. To monitor long-term antibody levels, a cohort of MMR-vaccinated individuals was established in 1982, and follow-up of the same individuals continued for over 30 years [14, 15]. The coverage of the first dose of MMR has remained consistently at or above 95% [16, 17]. Finland successfully eliminated endemic measles by the early 1990s [18, 19]. Since then, only sporadic imported measles cases with very limited secondary transmission have been reported [8, 20, 21], reflecting sustained herd immunity despite persistent low-level exposure.

An antibody concentration of 120 mIU/ml measured with the plaque reduction neutralizing test (PRNT) is widely considered a correlate of protection against measles [22–24]. Declining levels of measles-specific antibodies in vaccinated individuals [25] may be associated with reduced protection against measles infection. However, assessing the full extent of vaccine-induced protection is challenging as it involves both antibody-mediated and cell-mediated immune responses [2, 26]. Notably, the latter has been shown to persist longer [27, 28]. Moreover, there is evidence that vaccinated individuals who contract measles tend to experience milder symptoms [13, 29] and are less likely to transmit the virus [30, 31]. The long incubation period of measles (7–21 days post-exposure) may allow the immune system to limit viral replication and reduce infectiousness, even if infection is not completely prevented [2]. Indeed, sustained herd immunity reflects not only protection against infection but, more importantly, the interruption of transmission. Accordingly, we define the *firewall population* in this study as the population required to sustain herd immunity, comprising both individuals who are protected from infection and those who, if infected, are unlikely to transmit measles.

Sustained herd immunity indicates that the proportion of firewall population is sufficiently large to interrupt population-level transmission. Based on the absence of measles epidemics in Finland and utilizing previously collected antibody measurements from the Finnish MMR-vaccinated cohort, we infer the antibody threshold corresponding to the firewall population.

## Methods

Previously measured longitudinal data from the Finnish MMR-vaccinated cohort [14, 15] were utilized to estimate the rate of antibody decline over time since vaccination in a population with virtually no measles circulation. Because different laboratory assays had been used during the follow-up, measurements were aligned to ensure comparability.

For any candidate antibody threshold, we predicted the proportion of the Finnish population in 2022 exceeding that threshold by applying the estimated antibody decline among vaccinated individuals. This predicted 2022 proportion was then compared with the critical proportion of the firewall population required for herd immunity ($\:1-1/{\mathrm{R}}_{0}$). We assumed $\:{\mathrm{R}}_{0}=10-15$, corresponding to a critical proportion of approximately 90–93.3%. Given the sustained absence of measles epidemics in Finland, the 2022 proportion must therefore exceed 90–93.3%. This comparison indicates whether a given candidate antibody threshold is sufficient to interrupt transmission in 2022.

### The Finnish MMR-vaccinated cohort

The Finnish MMR-vaccinated cohort was established in 1982, as described in [14]. At study entry (age 14–18 months), the majority of participants (167 of 178) were seronegative for measles prior to receiving their first dose of MMR vaccine [14]. The second dose was administered at six years of age, following the national immunization schedule.

To estimate the rate of antibody decline over time, we analyzed follow-up data from the 167 initially seronegative participants after their second vaccine dose. Serum samples were collected during eight sampling rounds in 1987–2012, when participants were aged 6, 7, 10, 13, 16, 21, 26, and 31 years. Data from the last sampling round have not been previously reported.

The MMR cohort study has been conducted under varying legislative frameworks over the decades. It was last approved by the Hospital District of Helsinki and Uusimaa Ethics Committee of Epidemiology and Public Health in 2011 (340/13/03/00/2011). All vaccinees, or their parents, had given written consent before the samples were collected.

## Measurement of antibody concentration

For the first six sampling rounds (ages 6–21 years), measles antibody concentrations were measured using the *haemagglutination inhibition* (HI) assay, as previously described [14]. In the analysis, an HI titer of 1:5 was converted to 80 mIU/ml based on the IS2 [14, 32]. For the last three rounds (ages 21–31 years), antibody concentrations were determined using a commercial IgG *enzyme immunoassay* (EIA; Enzygnost, Dade Behring), which directly reports IgG concentrations in mIU/ml [15]. Additionally, at the fourth sampling round (age 13 years), the PRNT was also performed [14].

At the sixth sampling round, where both EIA and HI assays were performed, the geometric mean concentration (GMC) was higher with EIA (769 mIU/ml) than with HI (430 mIU/ml). Similarly, at the fourth round, the GMC measured by PRNT (1869 mIU/ml) was approximately three times that measured by HI (624 mIU/ml) [14]. To estimate the decline in antibody levels over time, it is essential to ensure compatibility across different laboratory assays and sampling rounds. For clarity, we denote the assay-specific units as mIU/ml[HI], mIU/ml[EIA], and mIU/ml[PRNT].

### Modeling the decline of antibody levels

To characterize the long-term dynamics of vaccine-induced antibody levels in the cohort following the second MMR dose, we employed a random effects model. The model disentangles the underlying concentration trajectory of each participant from its observed values measured at discrete sampling rounds.

Let $\:{a}_{1}$ be the age at the first sampling round, shortly after the second vaccine dose. For an individual * i*, the natural logarithm of underlying antibody concentration at age $\:a>{a}_{1}$ was modelled by

1 $$\:\begin{array}{c}{U}_{i}\left(a\right)={{\upmu\:}}_{U}+{U}_{i}-\beta\,\:{\log}\left(a-{a}_{1}+1\right)\end{array}$$.

Here, $\:{{\upmu\:}}_{U}$ is the mean antibody level in the cohort at age $\:{a}_{1}$, and

2 $$\:\begin{array}{c}{U}_{i}\sim\:N\left(0,{{\upsigma\:}}_{U}^{2}\right)\end{array}$$.

is an individual-specific random intercept capturing between-individual variability in antibody concentration. The coefficient $\:{\upbeta\:}$ quantifies the rate of decline in antibody levels over time. On a non-logarithmic scale, the antibody levels are proportional to

3 $$\:\begin{array}{c}1/{\left(a-{a}_{1}+1\right)}^{{\upbeta\:}}.\end{array}$$.

The expression was used to determine the half-life of antibody levels.

### Parameter estimation

To integrate data from both HI and EIA assays, used at 6–21 year and 21–31 year age ranges, respectively, we introduced a scaling factor $\:{e}^{{s}_{EIA}}$ to convert the units:

4 $$\:\begin{array}{c}1\hspace{0.17em}\mathrm{m}\mathrm{I}\mathrm{U}\mathrm{/}\mathrm{m}\mathrm{l}\mathrm{[}\mathrm{H}\mathrm{I}\mathrm{]}={e}^{{s}_{EIA}}\hspace{0.17em}\mathrm{m}\mathrm{I}\mathrm{U}\mathrm{/}\mathrm{m}\mathrm{l}\mathrm{[}\mathrm{E}\mathrm{I}\mathrm{A}\mathrm{]}.\end{array}$$

Additionally, for comparison with PRNT-based measurement, the conversion

5 $$\:\begin{array}{c}1\hspace{0.17em}\mathrm{m}\mathrm{I}\mathrm{U}\mathrm{/}\mathrm{m}\mathrm{l}\mathrm{[}\mathrm{H}\mathrm{I}\mathrm{]}=3\hspace{0.17em}\mathrm{m}\mathrm{I}\mathrm{U}\mathrm{/}\mathrm{m}\mathrm{l}\mathrm{[}\mathrm{P}\mathrm{R}\mathrm{N}\mathrm{T}\mathrm{]}\end{array}$$

was applied [14].

A joint probability model was constructed for the model parameters and the observed antibody concentrations, by assuming logistic measurement error distributions with scale parameters $\:{{\upsigma\:}}_{HI}>0$ and $\:{{\upsigma\:}}_{EIA}>0$ for measurements obtained via the HI and EIA assays, respectively (see Supplementary Material A.1, Eqs. (8–10)). The within-individual variation from the expected decline trajectory $\:\left(\mathrm{E}\mathrm{q}.1\right)$ was included in the measurement error component.

Independent flat priors were assigned to all model parameters to derive the posterior distribution of the parameters ($\:{{\upmu\:}}_{U}$, $\:{\upbeta\:}$, $\:{{\upsigma\:}}_{U}$, $\:{s}_{EIA}$, $\:{{\upsigma\:}}_{HI}$, $\:{{\upsigma\:}}_{EIA}$). Notably, the scaling parameter $\:{s}_{EIA}$ was estimated jointly with the other parameters, indicating that data from all sampling rounds, not just at age 21, contributed to the estimate.

The posterior distribution was explored using the Metropolis-Hasting algorithm. Further details on the joint probability model are provided in Supplementary Material A.1, and the performance of estimation is discussed in Supplementary Material B.

### Projected lifetime antibody levels

The posterior distribution of the model parameters was used to generate the posterior predictive distribution (PPD) of (logarithmic) antibody levels $\:{U}_{i}\left(a\right)$, as defined in $\:\left(\mathrm{E}\mathrm{q}.1\right)$, for all ages $\:a>{a}_{1}$, including ages beyond the maximum observed in the study cohort (Supplementary Material A.2). From the PPD, we numerically estimated (Supplementary Material A.3) the predicted proportion of vaccinated individuals whose measles antibody concentration exceeds a given threshold $\:{\uptau\:}$ at age $\:a>{a}_{1}$. When applying a threshold, $\:\mathrm{exp}\left({U}_{i}\left(a\right)\right)$ and $\:{\uptau\:}$ must be expressed in the same units throughout a single computation (e.g., mIU/ml[HI]).

### Firewall population under candidate antibody thresholds

For a range of candidate antibody thresholds $\:{\uptau\:}$ assumed capable of interrupting measles transmission, we predicted the corresponding proportion of the firewall population within the total Finnish population in 2022, ten years after the latest sampling round. A given candidate threshold was considered sufficient if the predicted firewall proportion exceeded the critical herd immunity level of 90-93.3%.

The total population was constructed as follows (for formulae, see Supplementary Material A.4). The estimation incorporated the realized vaccination coverage of 95% [17]. In the fully vaccinated age cohorts (ages 6–40 years), the estimated proportion of vaccinated individuals with antibody levels exceeding the threshold were considered part of the firewall population. The remaining vaccinated individuals, along with the unvaccinated, were assumed not to contribute to interruption of transmission. For simplicity, vaccinated children under age 6 were also assumed to be included in the firewall population, despite not having received the booster dose. For older age cohorts (> 40 years), the protective proportion ($\:{c}_{old})$ was set to 95% or 100% scenarios, reflecting bounds of lifetime measles exposure in the pre-vaccination era.

### Sensitivity analysis

Antibody levels typically decline most rapidly in the first years following vaccination, which may disproportionately influence the estimate of the decline parameter $\:{\upbeta\:}$. Base-case estimation used data from all eight sampling rounds. Given the specific interest in the long-term decline of antibody levels, we conducted a sensitivity analysis by re-estimating the model parameters using data from only the last five sampling rounds (ages 13–31 years). This approach, referred to as *tail-only estimation*, helps isolate the long-term trend from early post-vaccination dynamics.

## Results

### MMR cohort participants and available measurements

**Fig. 1:**
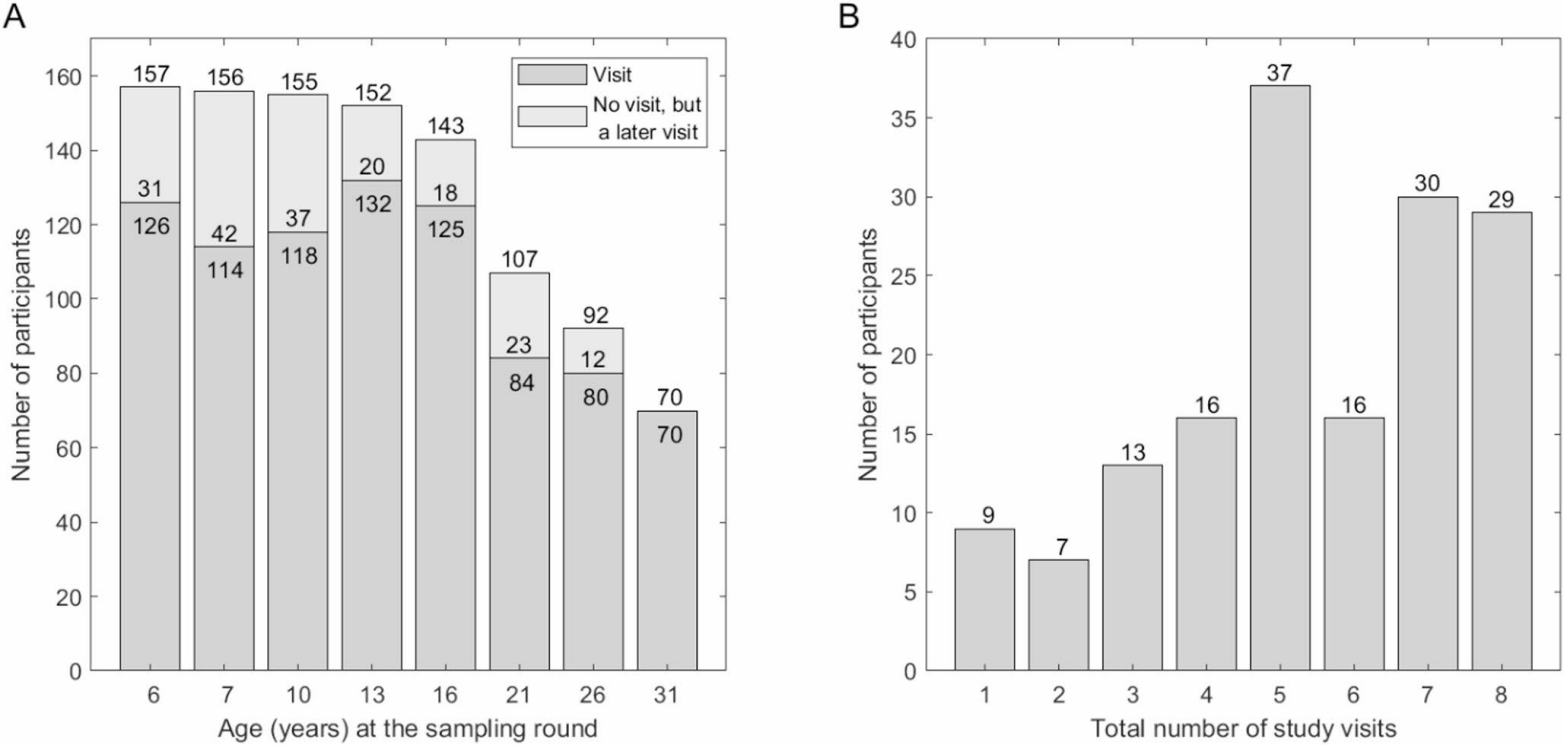
**A** The number of Finnish MMR cohort participants followed by sampling round. **B** Distribution of the total number of study visits per participant. **ALT TEXT**: Graphs showing visits of study participants, by sampling round and in total

A total of 157 out of 167 participants from the Finnish MMR-vaccinated cohort provided at least one serum sample during the follow-up from ages 6 to 31 years, following the administration of the second MMR dose at age 6. Among these participants, the mean follow-up duration exceeded 20 years. Continued participation was high, with 107 (68%), 92 (59%), and 70 (45%) individuals still contributing samples after ages 21, 26, and 31 years, respectively (Fig. 1A). In total, 849 samples were collected, representing 68% of the 1,256 potential visits (mean 5.4 visits per participant, Fig. 1B). All 84 samples collected at the 21-year sampling round had been analyzed using both the HI and EIA assays. For the sensitivity analysis, which focused on the last five sampling rounds (ages 13–31 years), measurements from 152 of the 157 participants were available in at least one of these rounds (Fig. 1A).

### Observed antibody concentration

**Fig. 2:**
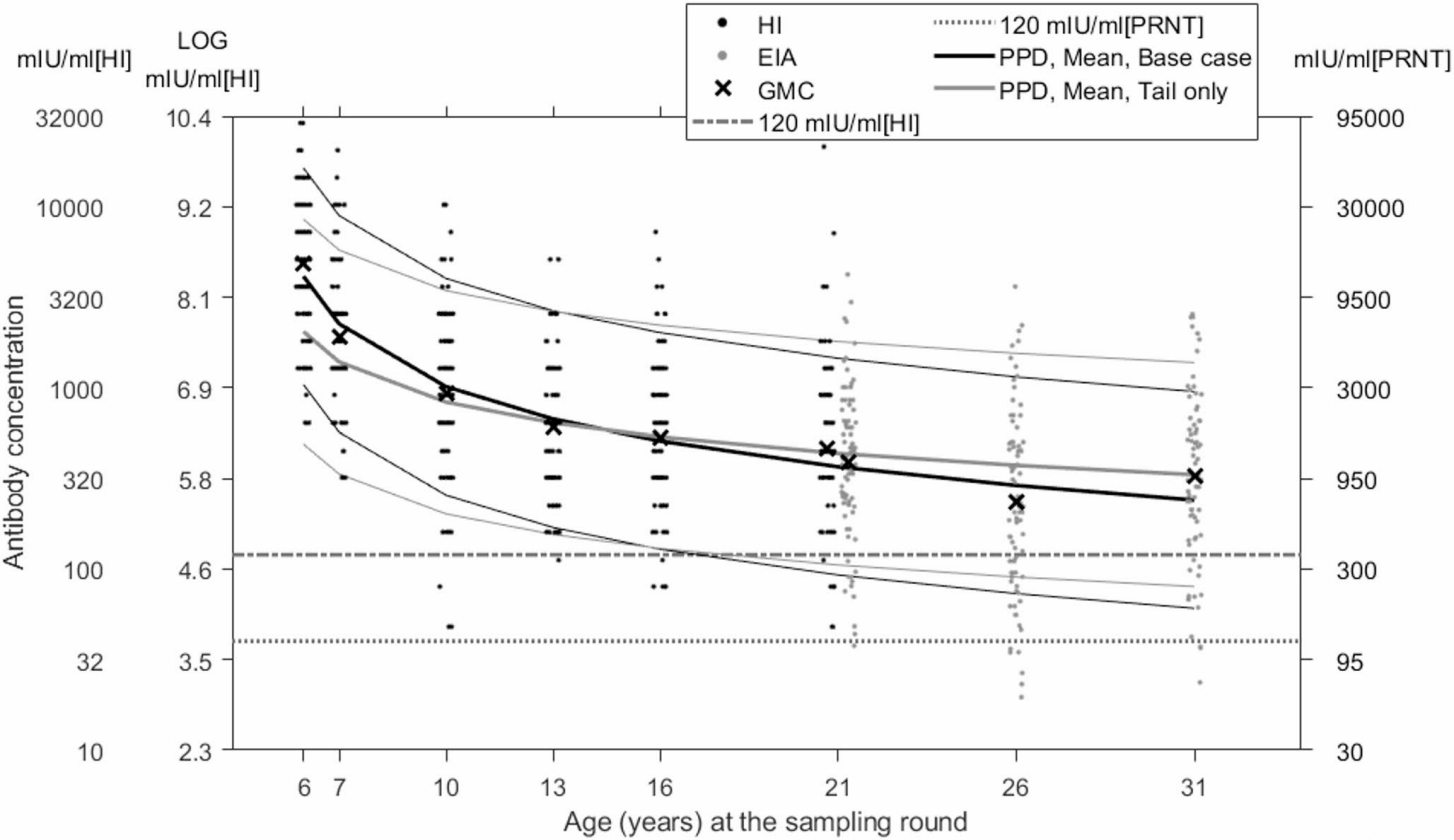
Measles antibody concentrations by sampling round (corresponding to age at sampling) and laboratory assay, with posterior estimates of antibody decline. Antibody concentrations are shown in three units: mIU/ml[HI] and its logarithm on the left y-axis, and mIU/ml[PRNT] on the right y-axis. For the first six sampling rounds (ages 6–21 years), concentrations were measured using the HI assay (black dots). For rounds 6–8 (ages 21–31 years), the EIA assay was used (gray dots), with results converted to HI-based units. The geometric mean concentration (GMC, **x**) is shown for each sampling round and assay. Posterior predictive distributions (PPDs) of antibody levels are presented for the base case estimation (black) and the tail-only estimation (gray), which includes only data from ages 13–31 years. Posterior means and 90% credible intervals (thin lines) are indicated. Horizontal lines mark antibody levels of 120 mIU/ml[HI] (dash-dot line) and 120 mIU/ml[PRNT] (dashed line). **ALT TEXT**: Graphs showing observed measles antibody concentrations across participant ages, and model fits to their decline

Observed antibody concentrations by sampling round are presented in Fig. 2, with values shown in both mIU/ml[HI] (left axis) and mIU/ml [PRNT] (right axis) for comparison. The commonly referenced threshold of 120 mIU/ml is presented in both scales. The discrete nature of HI titers, in contrast to the continuous EIA values, is evident in the figure (black vs. gray dots).

Antibody levels measured by the HI assay declined over time, from a GMC of 4859 mIU/ml[HI] shortly after the booster, to 430 mIU/ml[HI] at the 21-year sampling round. EIA-based measurements, converted to the HI scale for comparability (see below), showed a further decline from 390 mIU/ml[HI] at age 21 to 331 mIU/ml[HI] at age 31 (Fig. 2). In the later sampling rounds, an increasing proportion of samples fell below the 120 mIU/ml[HI] threshold, but only a few dropped below the threshold of 120 mIU/ml[PRNT] (right axis).

### Quantitative estimates of antibody decline

All model parameters were well identified (see Supplementary Material B). The model fit to the observed antibody concentrations is shown in Fig. 2.

The posterior mean of the initial mean antibody level $\:{{\upmu\:}}_{U}$ was 8.33, with a 95% credible interval (CI) of (8.18,8.48) (Table 1). This estimate closely aligns with the initial GMC of 4859 mIU/ml[HI], as log(4859) = 8.5 (Fig. 2). The estimated between-individual variability $\:{{\upsigma\:}}_{U}$ of was 0.83 (CI: 0.73–0.93), substantially higher than the estimated measurement error scales for the HI and EIA assays: $\:{{\upsigma\:}}_{HI}=0.21$ (CI: 0.20–0.23) and $\:{{\upsigma\:}}_{EIA}=0.31$ (CI: 0.27–0.35).

The scaling parameter $\:{s}_{EIA},$ used to convert EIA-based concentrations to the HI scale $\:\left(\mathrm{E}\mathrm{q}.4\right)$, was estimated at 0.69 (CI: 0.60–0.77). This corresponds to a conversion factor of $\:\mathrm{exp}\left(0.69\right)\approx\:2$, meaning that 2 mIU/ml[EIA] ≈ 1 mIU/ml[HI]. Examples of unit conversions are provided in Table 2.

In the base case, the estimated mean antibody decline parameter $\:{\upbeta\:}$ was 0.87 (CI: 0.84–0.90), Table 1. This corresponds to a first half-life (from 100% to 50%) of approximately 10 years, starting from age 13, the first data point in the tail-only estimation, and a second half-life (from 50% to 25%) of 22 years from age 23. In the tail-only sensitivity analysis, the estimated decline parameter $\:{\upbeta\:}$ was slower at 0.56 (CI: 0.44–0.67), corresponding to longer half-lives of 20 and 67 years, respectively.

**Table 1:** Posterior mean estimates and 95% posterior credible intervals (CIs) for the population-level parameters in the base case, using data from all sampling rounds (ages 6–31 years), and in the tail-only estimation, which includes data from only the last five sampling rounds (ages 13–31 years)

Parameter	*Base case*		*Tail-only estimation*	
	Mean	95% CI	Mean	95% CI
$\:{\upbeta\:}$	0.87	(0.84,0.90)	0.56	(0.44,0.67)
$\:{{\upmu\:}}_{U}$	8.33	(8.18,8.48)	7.61	(7.31,7.91)
$\:{{\upsigma\:}}_{U}$	0.83	(0.73,0.93)	0.85	(0.75,0.95)
$\:{{\upsigma\:}}_{HI}$	0.21	(0.20,0.23)	0.16	(0.15,0.18)
$\:{{\upsigma\:}}_{EIA}$	0.31	(0.27,0.35)	0.30	(0.26,0.34)
$\:{s}_{EIA}$	0.69	(0.60,0.77)	0.48	(0.38,0.58)

The MCMC sample size was 4000

**Table 2:** Conversions between HI-based, EIA-based and PRNT-based units for selected antibody concentrations

mIU/ml[HI]	mIU/ml[EIA]	mIU/ml[PRNT]
40	80	**120**
60	**120**	180
**120**	240	360

The conversion between mIU/ml[HI] and mIU/ml[EIA] is based on the estimate from this study. The conversion between mIU/ml[HI] and mIU/ml[PRNT] is based on geometric mean concentrations reported in [14].

### Projected lifetime decline of measles antibodies

**Fig. 3:**
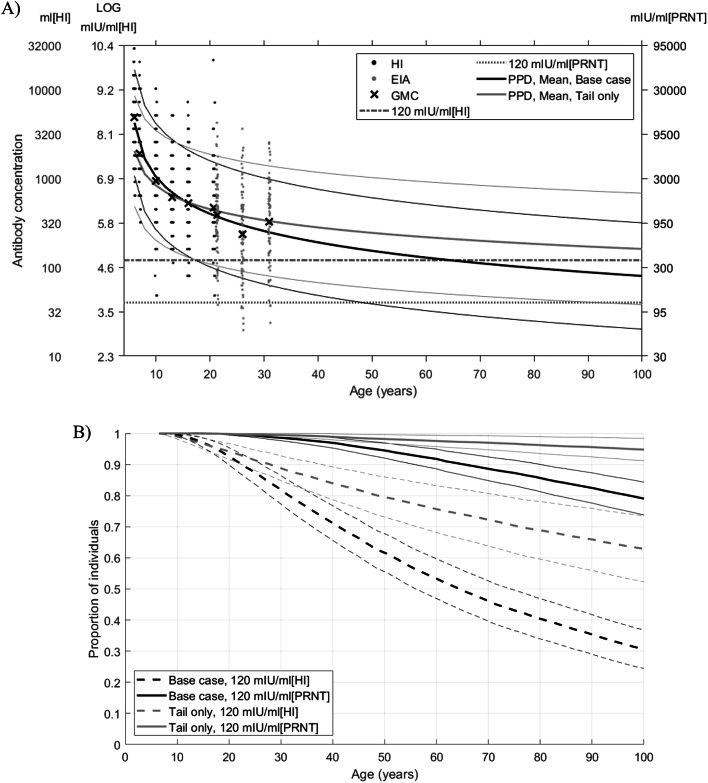
**A** Projected measles antibody levels based on the base case estimation (black) and the tail-only estimation (gray), extrapolated up to age 100 years. Mean estimates are shown as thick solid lines, and 90% credible intervals as thin lines. Horizontal lines mark antibody levels of 120 mIU/ml[HI] (dash-dot line) and 120 mIU/ml[PRNT] (dashed line). **B** Predicted age-specific proportions of MMR-vaccinated individuals with measles antibody levels exceeding thresholds of 120 mIU/ml[HI] (dashed lines) or 120 mIU/ml[PRNT] (equivalent to 40 mIU/ml[HI], solid lines), based on the base case estimation (black) and the tail-only estimation (gray). Thick lines represent means, and thick lines indicate 90% posterior uncertainties. **ALT TEXT**: Graphs showing model fits to measles antibody levels, extrapolated above participant ages, and proportions of individuals with antibody concentrations above specified thresholds

Figure 3A presents the posterior predictive antibody levels by age, projected up to 100 years, and Fig. 3B presents the predicted age-specific proportion of vaccinated individuals with measles antibody levels exceeding various assumed thresholds. In the base case, the proportion falls below 90% between ages 20 and 26 when using the 120 mIU/ml[HI] threshold, and drops below 70% between ages 36 and 47. With the lower threshold of 120 mIU/ml[PRNT] (approximately 40 mIU/ml[HI]), the proportion remains above 90% until ages 55 to 80 and stays above 70% thereafter. In the tail-only sensitivity analysis, where the antibody decline was estimated to be slower, the proportion exceeding the 120 mIU/ml[HI] threshold falls below 90% between ages 22 to 37, and below 70% between ages 56 and over 100. With the lower 120 mIU/ml[PRNT] threshold, the proportion remains above 90% across all ages.

### Year 2022 population-level protection by protective threshold against transmission

**Fig. 4:**
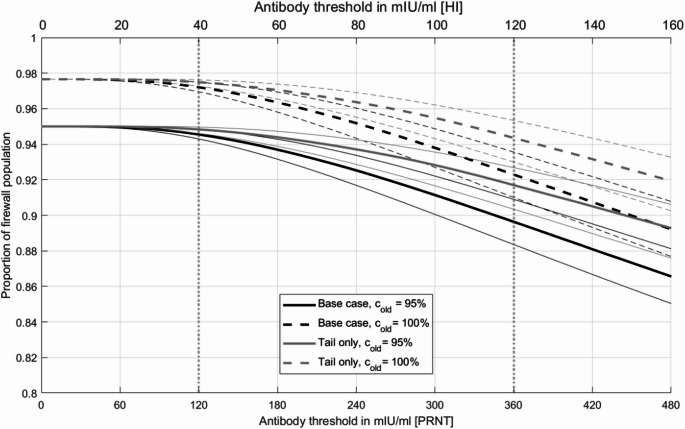
Predicted proportion of firewall population, individuals unable to transmit measles, in the Finnish population in 2022, as a function of the candidate antibody threshold (x-axis). Results are shown for the base case estimation (black) and the tail-only estimation (gray), assuming a protected proportion (c_old_) of 95% (solid line) or 100% (dashed line) among older unvaccinated cohorts with prior measles infection. The candidate threshold is presented in mIU/ml[HI] (top x-axis) and mIU/ml[PRNT] (bottom x-axis). Vertical lines highlight the levels of 120 mIU/ml[HI] and 120 mIU/ml[PRNT]. **ALT TEXT**: Graph showing the proportion of the firewall population under different model assumptions, plotted against varying antibody thresholds

Figure 4 presents the proportion of the firewall population (individuals exceeding a candidate antibody threshold) in the Finnish population in 2022 as a function of the threshold. The four scenarios reflect combinations of choices in parameter estimation (base case vs. tail-only estimation) and assumptions about immunity in the older population due to prior measles infection (95% vs. 100% protected).

Under a candidate antibody threshold of 120 mIU/ml[HI], the base-case estimation assuming 95% immunity in the older population resulted in a firewall population proportion of 88–90%, below the $\:{R}_{0}$-based critical proportion of 90–93.3% for herd immunity. In contrast, the tail-only estimation combined with 100% assumed immunity among older individuals resulted in a likely sufficient proportion of 94–96%. The two remaining scenarios produced intermediate estimates of 90–94%, close to the $\:{R}_{0}$-based critical proportion.

Under a candidate antibody threshold of 40 mIU/ml[HI], equivalent to 120 mIU/ml[PRNT] via the conversion $\:\left(\mathrm{E}\mathrm{q}.5\right)$, the firewall population proportion exceeded the R_0_-based critical proportion in all scenarios. Estimated proportions ranged from 94–95% assuming 95% immunity in older individuals ($\:{c}_{old}$ 95%) to 97–98% assuming full immunity ($\:{c}_{old}$ 100%), for both the base-case and tail-only estimations.

## Discussion

A gradually decelerating decline of measles antibody levels among vaccinated individuals, based on repeated measurements in the Finnish MMR-vaccinated cohort over a 25-year period following the second vaccine dose at age 6, combined with the continued absence of measles epidemics in Finland, indicates long-lasting vaccine-induced protection against measles transmission. Nevertheless, due to non-uniqueness in estimating the decline, we were unable to determine an unambiguous antibody threshold required to interrupt measles transmission.

Antibody levels were estimated to decline slower than exponentially. After an initial steep decline, the estimated half-life in the base case was 10 years from age 13 and 22 years from age 23 onward. When the early time points of steep decline were excluded (tail-only estimation, using data from age 13 onward), the decline appeared even slower, with estimated half-lives of 20 years from age 13 and 67 years from age 33.

The absence of measles epidemics in Finland more than 40 years into the MMR vaccination programme provides information about antibody levels sufficient to interrupt measles transmission. This analysis combines estimates of declining antibody levels, measured using HI and EIA assays, with vaccination coverage and assumptions regarding infection-induced immunity in older, unvaccinated cohorts. If the true rate of antibody decline corresponds to the trajectory of the base case estimation, the continued absence of measles outbreaks suggests that the antibody threshold required to interrupt measles transmission is likely well below 120 mIU/ml in HI-based units, close to the HI positivity cut-off of 80 mIU/ml [15]. Otherwise, the proportion of individuals capable of transmitting measles would likely be too high to prevent outbreaks. If the actual decline is slower, corresponding to the tail-only estimation, antibody levels remain above 120 mIU/ml for a substantially longer period. The basic reproduction number $\:{R}_{0}$ is not a universal constant, and including a relatively low value of R_0_ ≈ 10 in the analysis therefore makes the antibody threshold estimates conservative. While the analysis does not establish the precise mechanism, the sustained absence of measles epidemics in a highly vaccinated population reinforces the evidence for long-lasting vaccine-induced protection against measles transmission.

The rationale for using data from only the last five sampling rounds (ages 13–31 years) in the tail-only sensitivity analysis was to reduce the influence of the early, pronounced antibody decline on long-term projections. While the base case estimation already incorporated a declining waning rate, the tail-only estimation yielded an even slower long-term decline. This suggests that antibody levels may persist substantially longer than what would be inferred from the early post-vaccination pattern. Supporting this interpretation, a large Austrian retrospective seroprevalence study found that measles antibody levels in vaccinated cohorts remained largely stable beyond the youngest ages [4], aligning more closely with our tail-only estimates than base-case estimates.

Some signs of waning vaccine-induced immunity have been previously reported. A Spanish study covering measles cases from 2014 to 2020 found a higher proportion of vaccinated cases in older cohorts [12]. An Italian study reported that even up to 20% of vaccinated 22-year-old individuals lacked detectable anti-measles IgG, compared to 6% among those with prior infection [33]. Nevertheless, post-outbreak evaluations in France 2017–2019 [34] and in Germany 2014–2015 [35] estimated long-term vaccine effectiveness above 90% even in the oldest vaccinated cohorts. While protection against infection and transmission may differ, these findings on long-term vaccine effectiveness align with the present study’s estimates regarding protection against measles transmission.

A recent study [36] integrates population-level seroprevalence data with measles resurgences outside London to identify different models and antibody cut-offs consistent with the observed loss of herd immunity, i.e., it seeks an upper bound for the level of protection in the population. In contrast, our study applies Finland’s sustained herd immunity to infer a lower bound for protection.

The main strength of our study is the long-term follow-up of a permanent vaccinated cohort of the same individuals over 30 years, combined with a high participation rate. The absence of circulating measles virus means that the vaccinated individuals have not received natural immune boosting for decades. On the other hand, we cannot fully exclude the possibility that the antibody levels were influenced by exposure to measles prior to the second vaccine dose. If such exposure occurred, antibody levels in this cohort may be higher than in later birth cohorts who have lived entirely in a measles-free environment. Some degree of selection bias is also possible, for example in participation across different sampling rounds.

The primary methodological challenge was that antibody concentrations had been measured using different assays (HI, EIA, PRNT) at different times during the long follow-up. Despite calibration efforts of laboratory assays, the resulting antibody levels varied systematically at the cohort level, depending on the assay used. In general, a similar calibration-related uncertainty is present when data from multiple studies are combined, e.g. [25]. To facilitate the estimation of antibody decline over time in our study, conversion factors were applied between assay-specific units. EIA-based concentrations were approximately twice, and PRNT-based concentrations approximately three times (previously reported in [14]) of the corresponding HI-based values. While these conversions were internally valid within this study (see also [37]), they introduce uncertainty regarding absolute antibody concentration, particularly in relation to protective thresholds. Furthermore, even within a single assay, calibration may have differed between sampling rounds. Consequently, although our study identified qualitative relationships between antibody levels and population-level protection against transmission, it did not reveal a unique quantitative antibody threshold.

Different assays may not equally capture the functional properties of antibodies but instead offer complementary insights into immunity against measles [26]. Even very low levels of neutralizing antibodies may provide sufficient protection against measles infection. While non-neutralizing antibodies do not directly prevent infection, they may contribute to immune defense through other mechanisms and can influence factors like transmission.

## Conclusion

The decelerating decline in measles antibody levels among vaccinated individuals, coupled with the continued absence of measles outbreaks in Finland, supports the notion that the MMR vaccination with a 5-year interval offers durable protection against measles transmission. At the population level, maintaining high vaccination coverage thus appears to be a more pressing concern than the potential waning of immunity over time. Nevertheless, despite the evidence provided by the long follow-up time in this study, the possibility of a need for a measles booster vaccination at older ages cannot be entirely excluded.

## Supplementary Information

Below is the link to the electronic supplementary material.

Supplementary Material 1

Supplementary Material 2

## Data Availability

Data not publicly available.
