# Supplementary material for: Sustaining herd immunity against measles: Insights from a serological cohort study in an outbreak-free population
Source: Infection. 2026 May 27;54(4):2053–63. doi: 10.1007/s15010-026-02832-9 (PMC13476317; doi:10.1007/s15010-026-02832-9)
Supplement: Supplementary file 1 [file 15010_2026_2832_MOESM1_ESM.pdf]

A Supplementary methods

A.1 Joint probability model

Let a_j denote the age at the jth sampling round, $j = 1, \ldots, 8$. We thus have $(a_1, \ldots, a_8) = (6.5, 7.5, 10.5, 13.5, 16.5, 21.5, 26.5, 31.5)$.

For the numerical consideration, we assume that the decline formula (1),

$$U_i(a) = \mu_U + U_i + \beta \log(a - a_1 + 1), \quad U_i \sim N(0, \sigma_U^2), \tag{7}$$

is in the HI-based unit mIU/ml[HI].

The observed HI-based concentration $Y_i^{HI}(a_j)$ for individual i at age a_j, in mIU/ml[HI], is assumed to be distributed as

$$Y_i^{HI}(a_j) = U_i(a_j) + W_{ij}^{HI}, \tag{8}$$

where $W_{ij}^{HI} \sim \text{Logistic}(0, \sigma_{HI})$ is a logistic measurement error with scale parameter $\sigma_{HI} > 0$. Similarly, the observed EIA-based concentration $Y_i^{EIA}(a_j)$, in mIU/ml[EIA], is assumed to be distributed as

$$Y_i^{EIA}(a_j) = U_i(a_j) + s_{EIA} + W_{ij}^{EIA}, \tag{9}$$

with $W_{ij}^{EIA} \sim \text{Logistic}(0, \sigma_{EIA})$. Here, the term s_{EIA} is to convert U_i in mIU/ml[HI] into mIU/ml[EIA] by (4).

Recall that for $W \sim \text{Logistic}(\mu, \sigma)$, the probability density function is

$$f_W(w) = \frac{e^{-(w-\mu)/\sigma}}{\sigma(1 + e^{-(w-\mu)/\sigma})^2}. \tag{10}$$

In general, if $W \sim \text{Logistic}(\mu, \sigma)$, it holds $W + s \sim \text{Logistic}(\mu + s, \sigma)$.

For each individual i, $i = 1, \ldots, N$, all available measurements were used, with both HI-based concentration

$$Y_{ij}^{HI} = Y_i^{HI}(a_j), \quad j = 1, \ldots, 6,$$

and EIA-based concentration

$$Y_{ij}^{EIA} = Y_i^{EIA}(a_j), \quad j = 6, \ldots, 8.$$

Specifically, if the individual had a visit at round $j = 6$, at which both HI- and EIA-based values were available, they were both used in the estimation. For individual i, denote the set of indices j with available HI and EIA measurements by $J^{HI}(i)$ and $J^{EIA}(i)$, respectively.

The model unknowns consist of the individual random effects

$$U = (U_1, \ldots, U_N)$$

and the population-level parameters μ_U, σ_U, and β for antibody levels, and $\sigma_{HI}, \sigma_{EIA}$, and s_{EIA} for the measurements. We combine the six parameters to a vector θ,

$$\theta = (\mu_U, \sigma_U, \beta, \sigma_{HI}, \sigma_{EIA}, s_{EIA}).$$

Given the observed data $y = (y_1, \ldots, y_N)$ of all individuals,

$$y_i = \left((Y_{ij}^{HI})_{j \in J^{HI}(i)}, (Y_{i,j}^{EIA})_{j \in J^{EIA}(i)}\right),\ i = 1, ..., N,$$

the joint probability model of the observations and the model parameters is

$$\begin{aligned} p(y, U, \theta) = \prod_{i=1}^{N} \ & p(U_i|\sigma_U)p(\theta) \\ & \left\{ \prod_{j \in J^{HI}(i)} p(y_{ij}^{HI}|U_i, \mu_U, \beta, \sigma_{HI}) \right\} \\ & \left\{ \prod_{j \in J^{EIA}(i)} p(y_{ij}^{EIA}|U_i, \mu_U, \beta, \sigma_{EIA}, s_{EIA}) \right\}. \end{aligned} \tag{11}$$

The three conditional densities in (11) are those of (7), (8), and (9), respectively. Independent flat priors were used for all model parameters:

$$p(\theta) = p(\mu_U)p(\sigma_U)p(\beta)p(\sigma_{HI})p(\sigma_{EIA})p(s_{EIA}).$$

The priors for the parameters $\mu_U, \sigma_U, \sigma_{HI}, \sigma_{EIA}$ were restricted by a lower bound of 0 to ensure they are positive. This means using an improper uniform prior on $(0, \infty)$, on the log scale. Moreover, the parameters β and s_{EIA} were assigned a uniform prior on $(-\infty, \infty)$. Eventually, the posterior density for of the model unknowns is proportional to the joint density (11), i.e.,

$$p(\theta, U|y) = \frac{p(y, U, \theta)}{p(y)} \propto p(y, U, \theta). \tag{12}$$

A.2 Posterior predictive distribution

The posterior distribution (12) generates a posterior predictive distribution (PPD) for the antibody levels $U_i(a)$ at age a as

$$p(U_i(a)|y) = \int p(U_i(a)|\beta, \mu_U, \sigma_U)p(\beta, \mu_U, \sigma_U|y)d\beta d\mu_U d\sigma_U, \tag{13}$$

where by (7) the conditional antibody level is distributed by

$$U_i(a)|(\beta, \mu_U, \sigma_U) \sim N(\mu_U + \beta \log(a - a_1 + 1), \sigma_U^2). \tag{14}$$

The PPD (13) can be extrapolated beyond the maximum age of the study cohort.

A.3 Protected proportion with given threshold

If τ is a seroprotective threshold of antibodies against measles, the proportion of vaccinated individuals that are protected at age a is, by (13),

$$P(U_i(a) > \tau|y) = \int P(U_i(a) > \tau|\beta, \mu_U, \sigma_U)\, p(\beta, \mu_U, \sigma_U|y)d\beta d\mu_U d\sigma_U. \tag{15}$$

Because the conditional $U_i(a)|(\beta, \mu_U, \sigma_U)$ follows the normal distribution (14), the first part in the integrand is

$$P(U_i(a) > \tau|\beta, \mu_U, \sigma_U) = 1 - \Phi\left(\frac{\tau - \mu_U - \beta \log(a - a_1 + 1)}{\sigma_U}\right).$$

where Φ is the cumulative distribution function of the standard normal distribution. The integral (15) can thus be determined as a Monte Carlo average using numerical samples from the posterior distribution of model parameters, also beyond the ages of test rounds in this study. Indeed, the quantity $P(U_i(a) > \tau|y)$ in (15) can be interpreted as a probability, which already includes the uncertainty related to the model parameters through the posterior distribution, or, the expected proportion.

A.4 Population-level protection with given threshold

Next, the protection probability (15), with a given protective threshold τ, is extended to determine the proportion of susceptible individuals in the Finnish population in the year 2022. To this end, use the following notation:

- Let $P(a)$ be the number of individuals of age a (years), and denote the total population by
$$P_{all} = \sum_{a=0,1,2,\ldots} P(a).$$
- Let $q(a,\tau) = P(U_i(a) > \tau | y)$ be the proportion of vaccinated individuals that are protected at age a, over the age 6 years of the booster dose:
- Let $c(a)$ be the coverage of MMR vaccination at age a. The value $c(a) = 0.95$ is used for all vaccinated cohorts.
- Let $a_{vc} = 40$ be the upper age limit for the MMR vaccinated cohorts at year 2022, and assume that for ages $a > a_{vc}$, the proportion c_{old} is protected by experienced measles infection. The values $c_{old} = 1$ and $c_{old} = 0.95$ are used.
- For simplicity, as young children are not in the scope of this work, the vaccinated children are assumed to be protected also before the booster dose.

Then, for a given threshold τ, the proportion of susceptible population is

$$S(\tau) = \frac{1}{P_{all}} \cdot \left\{ \begin{array}{ll} \displaystyle\sum_{a=0}^{6} P(a) \cdot (1 - c(a)) & \text{before the 2nd dose} \\ \displaystyle + \sum_{a=7}^{a_{vc}} P(a) \cdot [\ (1 - c(a)) & \text{not vaccinated} \\ \qquad\qquad + c(a)(1 - q(a,\tau))\ ] & \text{vaccinated, not protected} \\ \displaystyle + \sum_{a>a_{vc}} P(a) \cdot (1 - c_{old}) & \text{older population.} \end{array} \right\} \quad (16)$$

The susceptible proportion (16) is estimated with a large range of protective thresholds τ.
