# Supplementary material for: Sustaining herd immunity against measles: Insights from a serological cohort study in an outbreak-free population
Source: Infection. 2026 May 27;54(4):2053–63. doi: 10.1007/s15010-026-02832-9 (PMC13476317; doi:10.1007/s15010-026-02832-9)
Supplement: Supplementary file 2 [file 15010_2026_2832_MOESM2_ESM.pdf]

B Supplementary Material Performans of Estimation

Disclaimer: Please note that the units and notation used in this section do not fully align with those presented in the main text. These were developed earlier and may differ from the final conventions used throughout the document.

In this supplement, we summarise the posterior distribution of the population-level parameters and analyze the convergence of the HMC sampling routine (Section B.1). In addition, we estimate the conversion factor s_{EIA} based on data from the one sampling round at which observations were made using both antibody assays (Section B.2). We assess the model fit by comparing model predictions to the actually observed data (Section B.3). Finally, we report a simulation study which was conducted to validate the implementation of the sampling algorithm and to assess the identifiability of the model parameters (Section B.4).

B.1 Posterior estimation

Statistical inferences were based on a numerical sample from the joint posterior distribution of the unknown model quantities (model parameters), realized through a Hamiltonian Monte Carlo (HMC) algorithm and implemented in Stan software. Four independent chains, each of 2000 samples, were run. The first 1000 samples from each chain were discarded as warm-up samples and the remaining 4000 samples were jointly retained for statistical inferences.

Table S1 summarises the marginal posterior distributions of the population-level parameters: antibody decline (β), initial mean log antibody level (μ_U), the standard deviation of the log antibody level (σ_U), conversion factor (s_{EIA}), and the scale parameters in the observation model ($\sigma_{HI}, \sigma_{EIA}$). The results for both the baseline and sensitivity analyses of the long-term antibody decline are presented.

Table S1: Posterior estimates of the population-level parameters in the baseline and sensitivity analyses. The baseline analysis was used data from all 8 sampling rounds while the sensitivity analysis employed data from the last 5 sampling rounds only. In both models, the posterior inferences were based on an HMC sample of size 4000. CI means the posterior probability interval (credible interval).

	Baseline				Sensitivity analysis			
Parameter	Mean	95% CI	n_{eff}	$\hat{R}$	Mean	95% CI	n_{eff}	$\hat{R}$
β	-0.87	(-0.90,-0.84)	4539	1.00	-0.56	(-0.67,-0.44)	2631	1.00
μ_U	5.56	(5.41,5.71)	305	1.01	4.84	(4.54,5.14)	1225	1.00
σ_U	0.83	(0.73,0.93)	600	1.01	0.85	(0.75,0.95)	703	1.01
σ_{HI}	0.21	(0.20,0.23)	4164	1.00	0.16	(0.15,0.18)	2684	1.00
σ_{EIA}	0.31	(0.27,0.34)	5188	1.00	0.30	(0.26,0.35)	4882	1.00
s_{EIA}	3.46	(3.37,3.54)	5145	1.00	3.25	(3.15,3.35)	3230	1.00

Apart from visual inspection of the HMC chains, the convergence of the sampling algorithm was assessed through the Gelman-Rubin diagnostic $\hat{R}$ and the effective sample size n_{eff} (Table S1) [1, 2]. Here $\hat{R}$ is the potential scale reduction factor and estimates how much the scale of the posterior distribution of each parameter might be reduced for an infinite number of sampling iterations. Strictly speaking, values below 1.05 are considered to be good [2]; however, generally, values below 1.1 are also considered satisfactory [1].

Moreover, n_{eff} is a crude measure of the effective sample size for each parameter [3]. It is an estimate of the number of independent samples with the same estimation power as the $N = 4000$ auto-correlated samples. Note that the effective sample size can be greater than the number of iterations if draws are anti-correlated because of the no-U-turn sampling (NUTS) algorithm used in Stan [3]. The effective sample size of each parameter in our model is substantial ($n_{eff} > 100$ and $\frac{n_{eff}}{N} > 0.001$) [1],[2]. It is worth noting that the hierarchical parameters (μ_U and σ_U) which usually have small n_{eff} because of their constrained geometries that make it difficult for the sampler to explore, also met this criterion.

Figures Figure S1 and Figure S2 show the pairwise posterior correlations between the population-level parameters in the baseline (all 8 sampling rounds) and sensitivity (only the last 5 sampling rounds) analyses. In the sensitivity analysis case, there is a strong negative correlation between the parameter

governing the antibody decline (β) and initial antibody level (μ_U), and also between β and conversion factor s_{EIA}. Not utilizing the first three rounds of data in the sensitivity analysis makes the initial antibody level (μ_U) more uncertain, which in turn translates to estimates of the rate of decline.

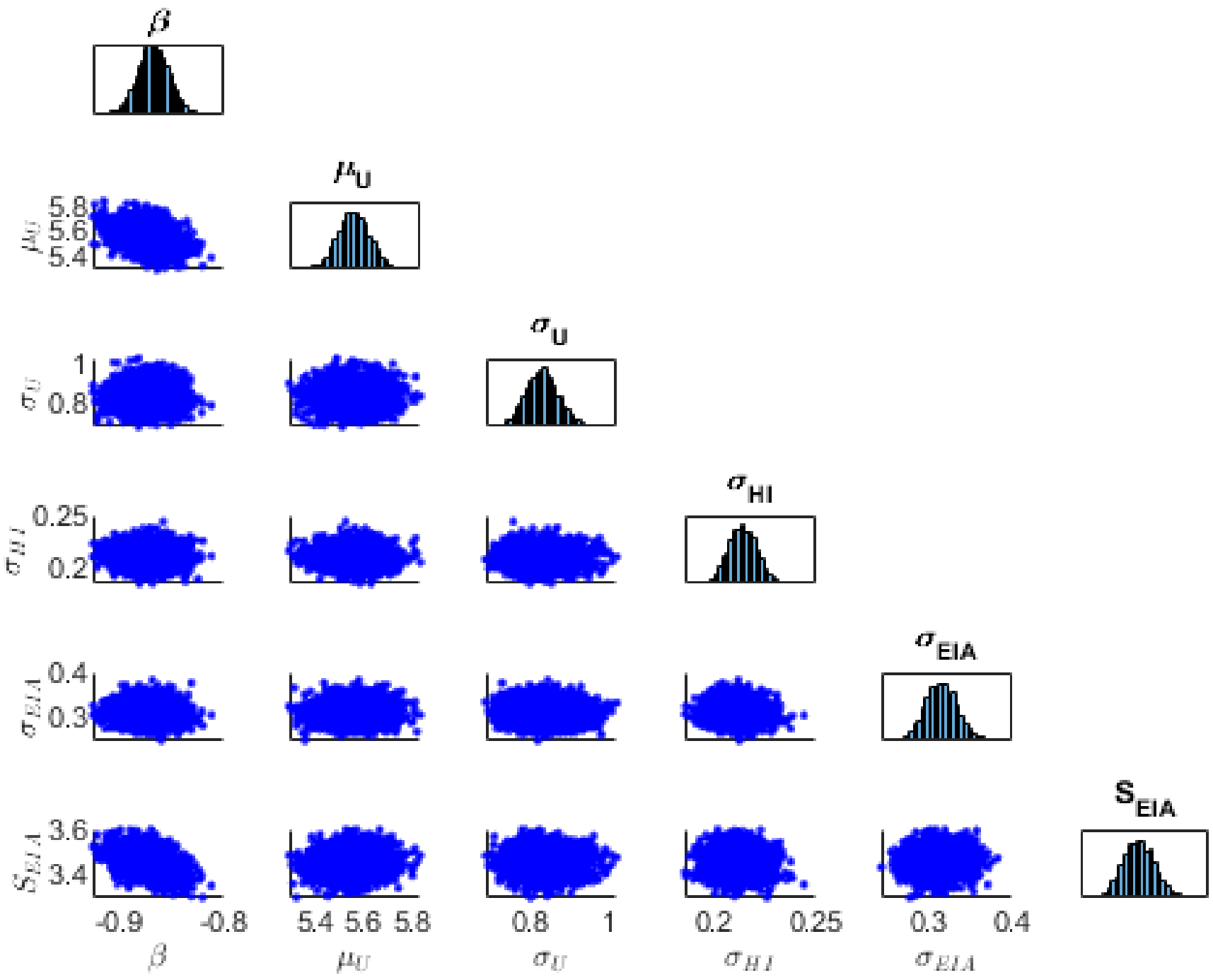

Figure S1: Pairwise marginal posterior distributions of the population-level parameters. The distributions are based on 4000 samples from the joint posterior distribution of all unknown model quantities. The analysis is based on using data from all 8 sampling rounds

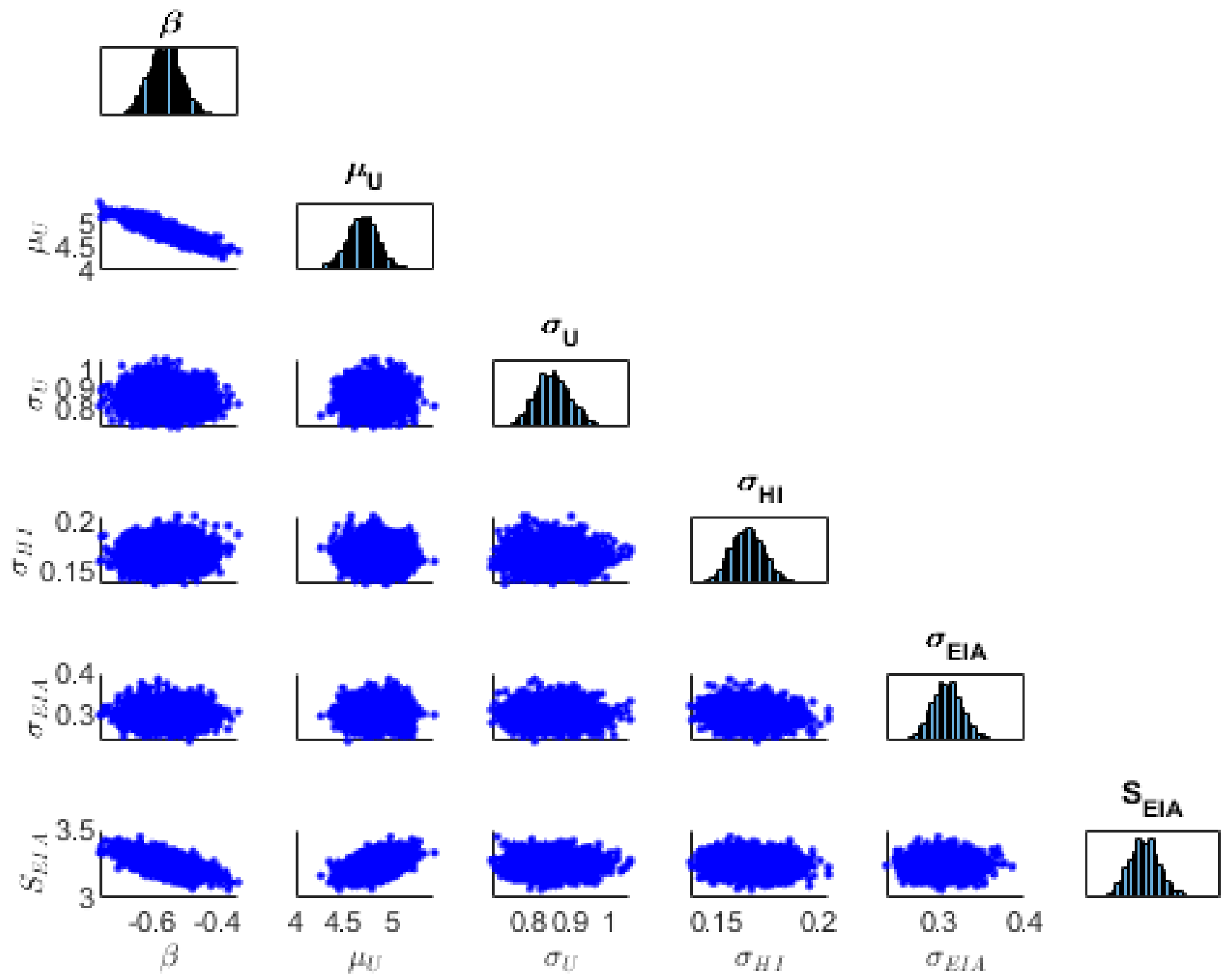

Figure S2: Pairwise marginal posterior distributions of the population-level parameters. The distributions are based on 4000 samples from the joint posterior distribution of all unknown model quantities. The analysis is based on using data from the five latest sampling rounds only.

B.2 Bridging between the two antibody assays – an additional analysis

To match the EIA-based antibody levels (ℓ) with the HI titers (h), the model include a multiplicative scaling factor $e^{s_{EIA}}$ (see Equation 1 in the main text). If the individual had a visit at test round $j = 6$, at which both HI titers and EIA-based measurements were available, both were used in the estimation of the model parameters. The posterior mean estimate of s_{EIA} was then 3.46 (95% CI 3.37,3.54; Table S1). However, to check how robustly s_{EIA} could be learned, we made a separate analysis and estimated s_{EIA} using only the data from test round $j = 6$. We then specified the model as

$$y_i^{EIA}(a_{j=6}) = s_{EIA} + \theta y_i^{HI}(a_{j=6}).$$

In this separate analysis, we obtained roughly the same posterior mean (3.65; 95% CI 3.24,4.07). The R^2 value for this linear model was 0.72 with a corresponding good fit of the model with the data.

Furthermore, we re-estimated the entire model, this time drawing values of s_{EIA} from the posterior of the simple model above (i.e. not estimating s_{EIA} with the rest of the parameters). The overall results of the model remained similar to the model where s_{EIA} was estimated along with other parameters.

B.3 Goodness of fit check

Having fitted a regression model to the observations, it is usual to check the validity of the underlying assumptions, including those made about the distribution of the residuals. In the Bayesian context, a typical approach is to calculate the so called Bayesian p values and show the corresponding comparisons visually [1]. Based on an HMC sample of size N from the posterior of the model unknowns $(\mu_{Uk}, \sigma_{Uk}, \beta_k, \sigma_{HIk}, \sigma_{EIAk}, s_{EIAk}, U_{ik})$, $k = 1, \ldots, N,$, $i = 1, \ldots, 164$, the Bayesian P value can be easily approximated as follows:

$$p = \frac{1}{N}\frac{1}{164}\frac{1}{8}\sum_{k=1}^{N}\sum_{i=1}^{164}\sum_{j=1}^{8}\mathbf{1}\Big\{r(\mathbf{y}_{ij}, \mu_{Uk}, \sigma_{Uk}, \beta_k, \sigma_{HIk}, \sigma_{EIAk}, s_{EIAk}, U_{ik}) \qquad \text{(S1)}$$
$$> \bar{r}(\mathbf{y}_{ijk}^{(rep)}, \mu_{Uk}, \sigma_{Uk}, \beta_k, \sigma_{HIk}, \sigma_{EIAk}, s_{EIAk}, U_{ik})\Big\}.$$

Here $\mathbf{1}(\cdot)$ is an indicator function and $r(\mathbf{y}_{ij}, \mu_{Uk}, \sigma_{Uk}, \beta_k, \sigma_{HIk}, \sigma_{EIAk}, s_{EIAk}, U_{ik})$ is the actual error calculated as the difference between the observed antibody titer and the predicted (individual-specific) mean titer, and $\bar{r}(\mathbf{y}_{ijk}^{(rep)}, \mu_{Uk}, \sigma_{Uk}, \beta_k, \sigma_{HIk}, \sigma_{EIAk}, s_{EIAk}, U_{ik})$ is a synthetic residual generated from the logistic distribution using the posterior parameter samples. If the model is appropriate, the P value should be around 0.50, or not at least close to zero or one.

Visually, one can plot samples $\Big(r(\mathbf{y}_{ij}, \mu_{Uk}, \sigma_{Uk}, \beta_k, \sigma_{HIk}, \sigma_{EIAk}, s_{EIAk}, U_{ik}), \bar{r}(\mathbf{y}_{ijk}^{(rep)}, \mu_{Uk}, \sigma_{Uk}, \beta_k, \sigma_{HIk}, \sigma_{EIAk}, s_{EIAk}, U_{ik})\Big)$, $k = 1, \ldots, N$, $i = 1, \ldots, 164$, $j = 1, \ldots, 8$ in a two-dimendional plot. If the model fit is good, approximately a half of the points should lie above the diagonal. Alternatively, a plot of the empirical and that of the theoretical probability density function (pdf) can be made to check their mutual agreement.

The residuals were computed for each measurement time of each individual for the goodness of fit analysis, based on the 4000 HMC samples realized from posterior distribution of the model parameters. In addition to the logistic measurement error model, we present here results also for two other distributions, i.e. the normal and extreme value (EV) distributions.

It can be seen from Figures S3-S5 that the logistic model provides a better fit as compared to the the normal and EV distribution. The scatter plot in Figure S6(a) shows that the real data residuals from the logistic distribution are half of the times larger than the simulated residuals, and the other way around. Whereas Figure S3(b)-c shows that for the case of the normal and EV distributions, the majority of the points are on the lower side of the diagonal (i.e. the simulated residuals are most of the time smaller than the actual residuals.)

The plots of the empirical and theoretical pdf of the residuals in Figure S4 show that both the tails and the peak fit better in the logistic case as compared to the case of the normal and EV distributions. Thus, for the case of the logistic distribution, only a smaller proportion of the empirical error does not conform with the same distribution as the theoretical residual as compared to the normal and EV distribution cases.

Again the plot of the simulated cumulative distribution function (CDF) of the residuals generated from the three distributions (Figure S5, also suggests that the empirical CDF of the actual residuals align better with that of the

synthetic residuals for the logistic distribution when compared to that of the EV and normal distribution.

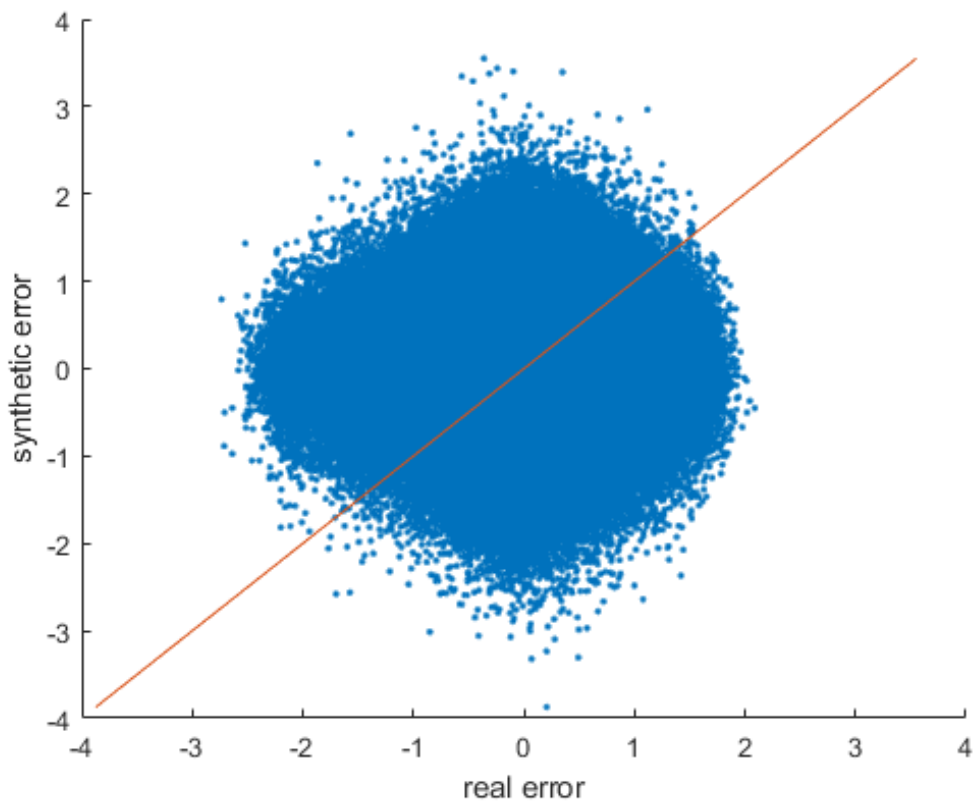

(a)

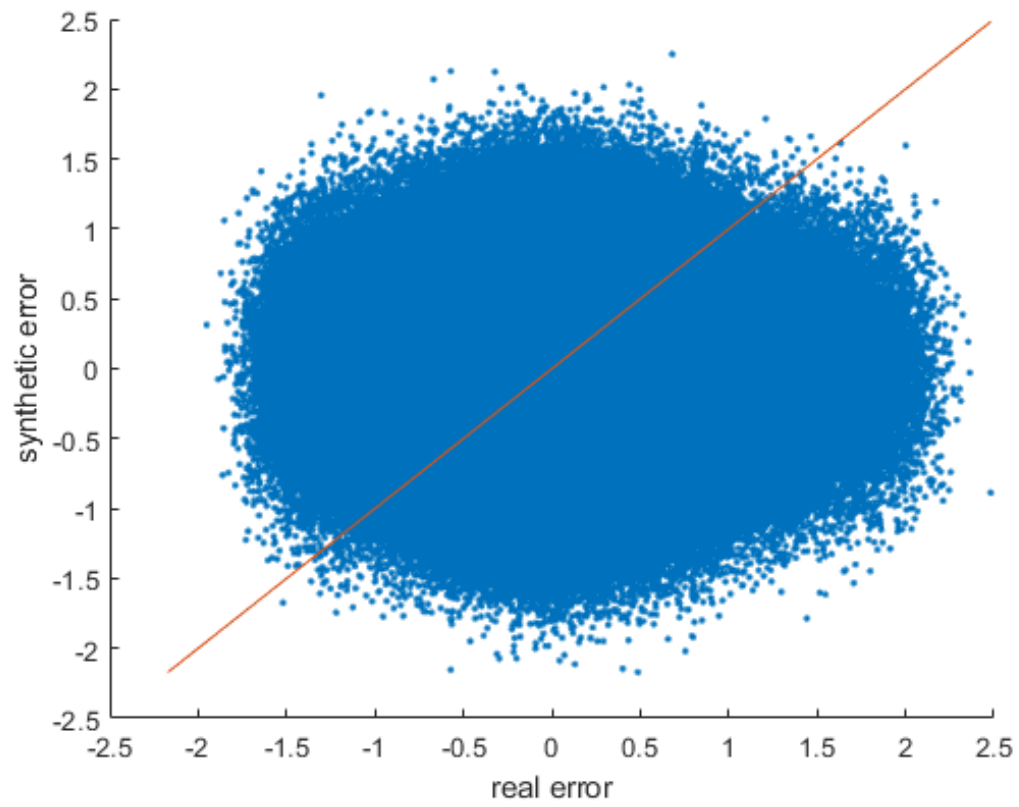

(b)

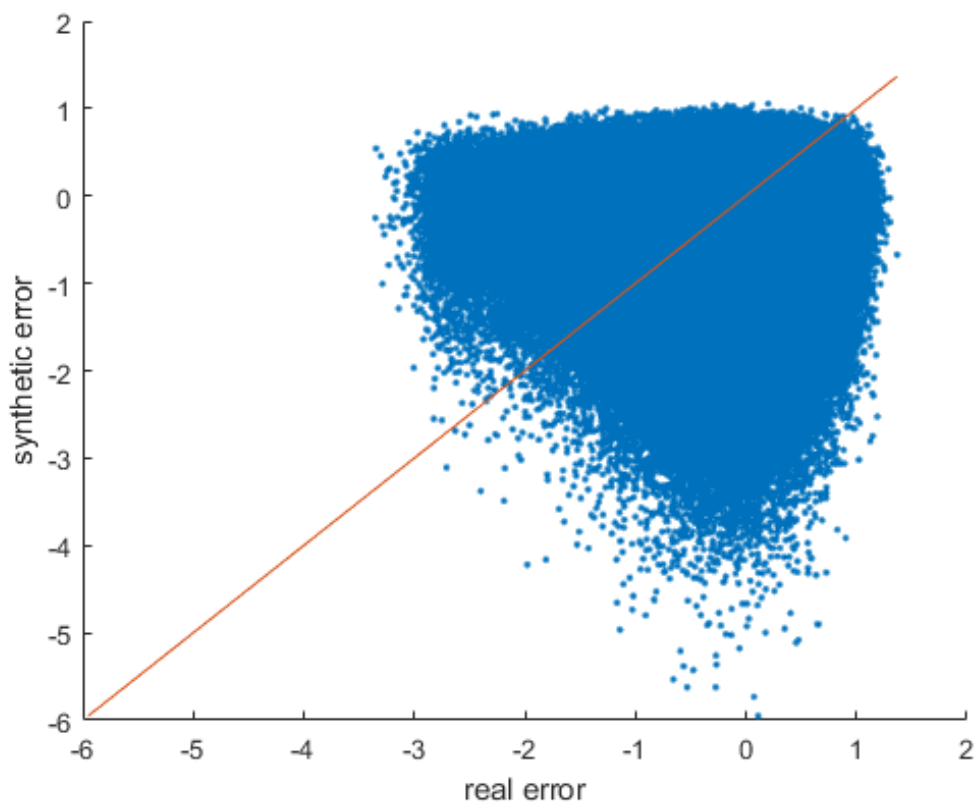

(c)

Figure S3: **Scatter plots for (a) logistic distributed actual and synthetic residual; P-value=0.4998 (b)Normal distributed actual and synthetic residual; P-value=0.4976 (c) (b) EV distributed actual and synthetic residual; P-value=0.4937.**

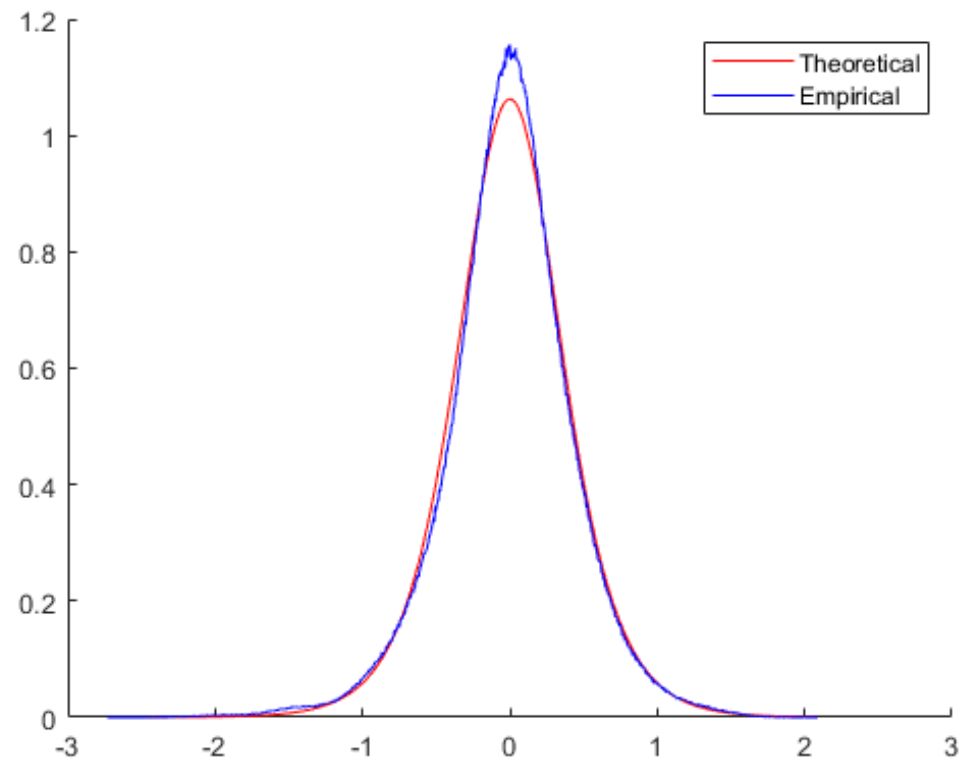

(a)

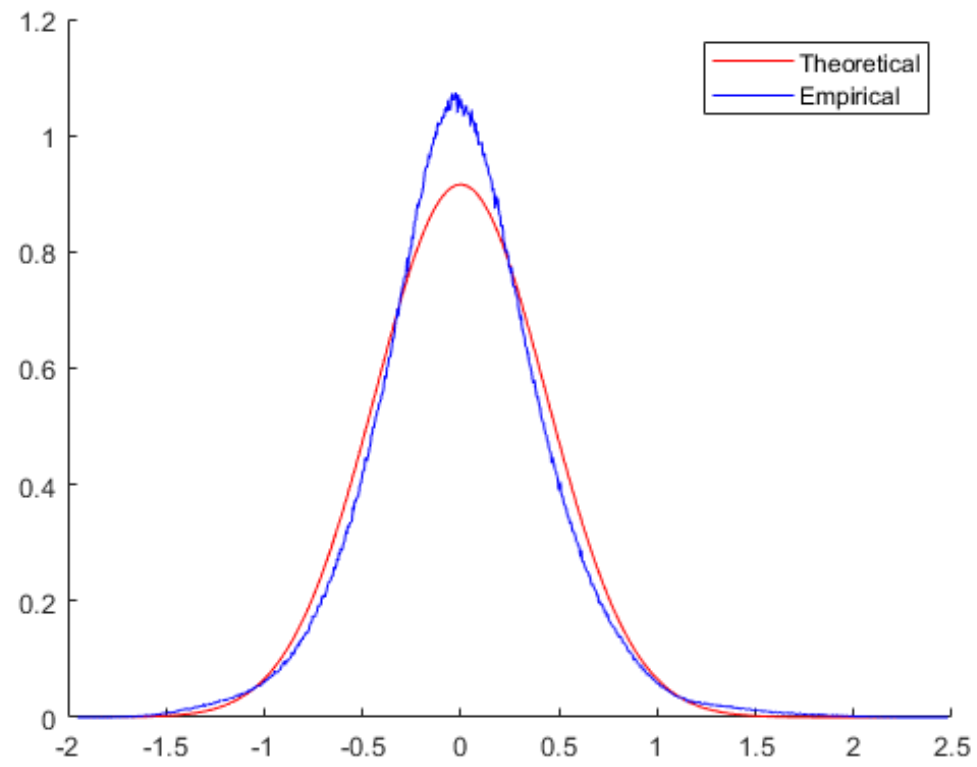

(b)

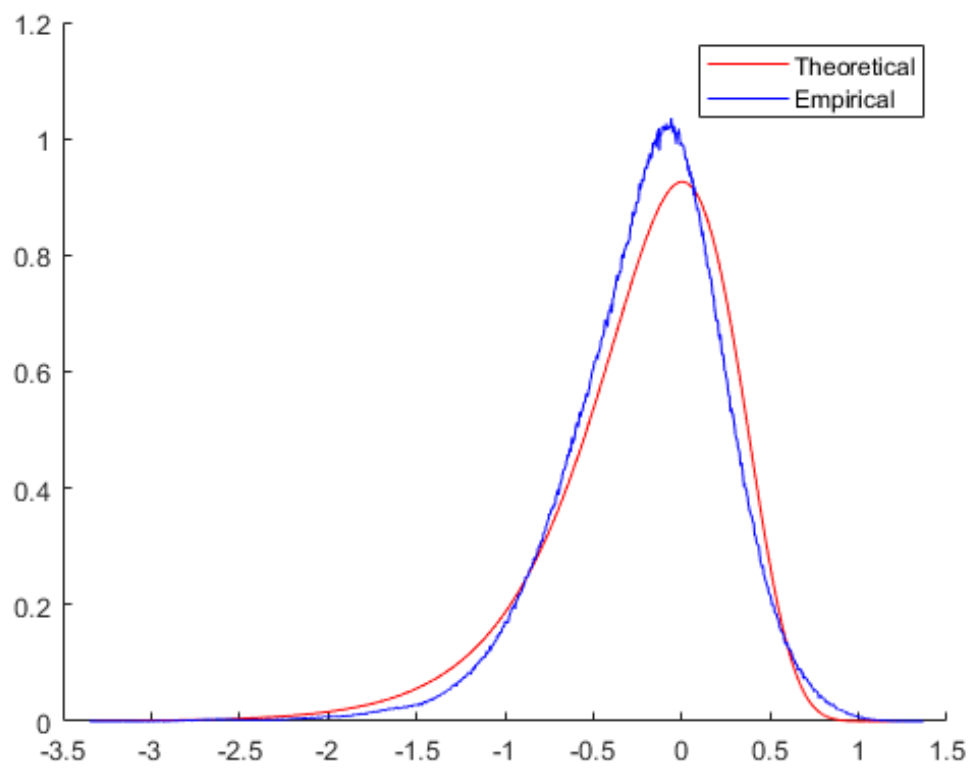

(c)

Figure S4: **Empirical and theoretical pdf plots for (a) logistic distributed residual (b)Normal distributed residual (C)EV distributed residual.**

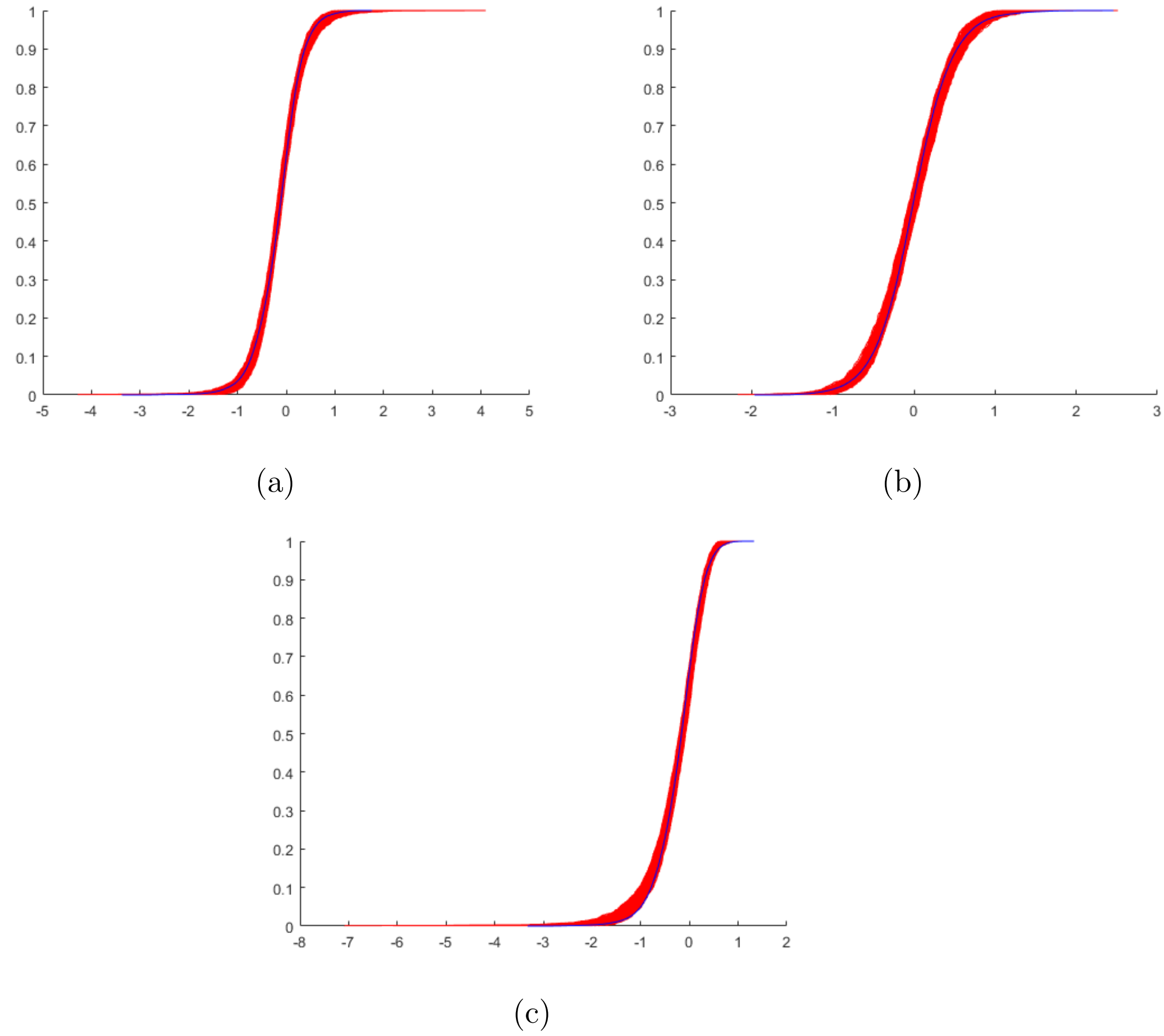

(a) (b) (c)

Figure S5: **Empirical cdf plots for the actual residuals(blue) and the synthetic residuals(red) for (a) logistic distribution (b)Normal distribution (c)EV distribution.**

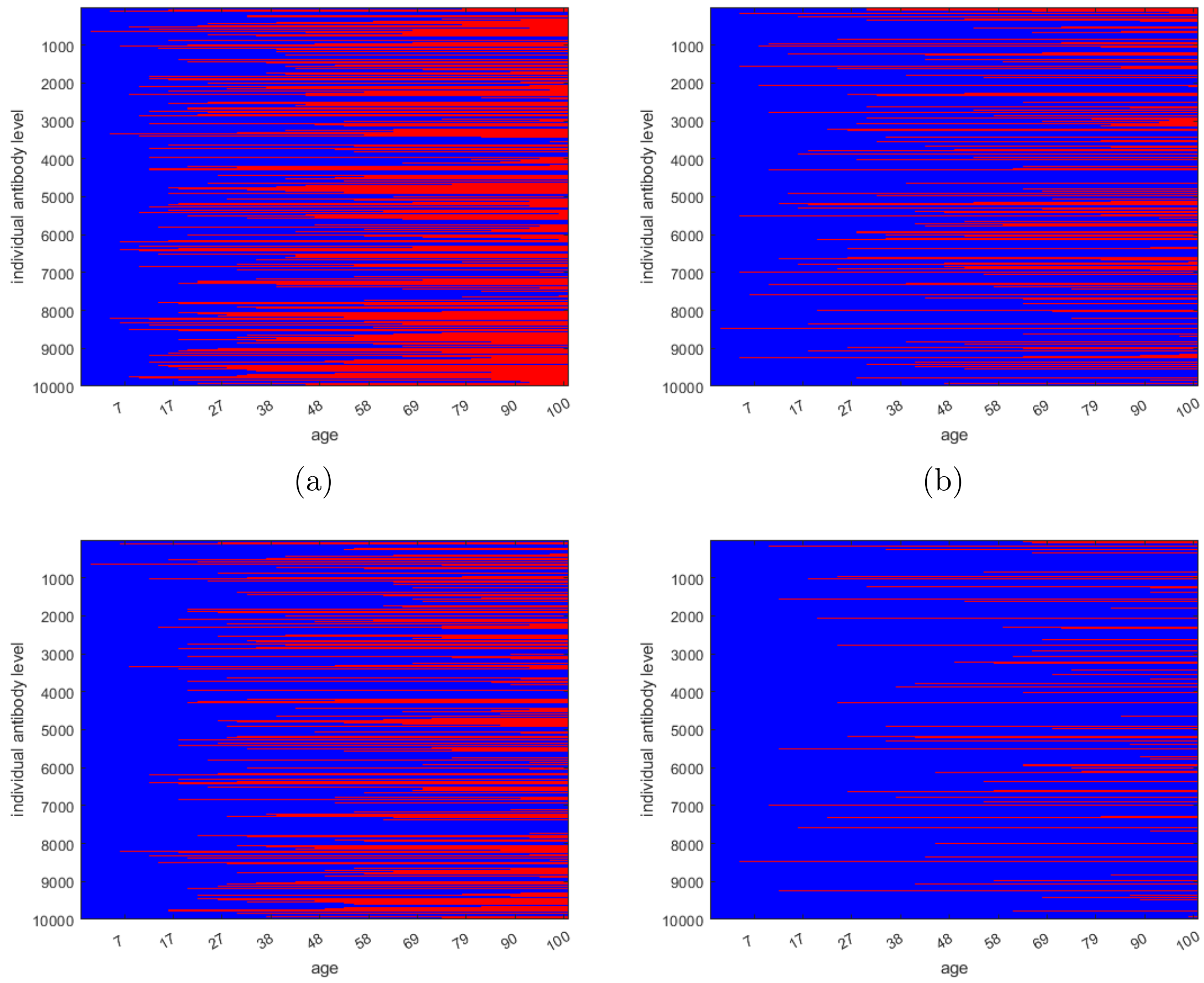

(a) (b) (c) (d)

Figure S6: **Colormap for 10000 generated individual antibody levels exceeding (blue) and not exceeding (red) the threshold of 120 mIU/ml ((a)-(b)) and 80 mIU/ml ((c)-(d)) with age, for the baseline analysis ((a) and (c)) and sensitivity analysis ((b) and (d)).**

B.4 Simulation Study

Given the small number of measurements in the available dataset, it is necessary to consider how the size of the data affects the uncertainty related to parameter estimation and the identifiability of the model parameters. This analysis will be useful in suggesting the number of measurements needed for better precision in this kind of study, in the future. Data were simulated from models given by Equation 4 with parameters ($\mu_U = 200, s_{EIA} = 15, \beta = -10, \sigma_U = 1, \sigma_{HI} = 4, \sigma_{EIA} = 0.5$). Two datasets of $N = 100$ individuals were simulated, first with 50 measurements per individual (30 measurements for the first set and 20 measurements for the second set), and second with eight measurements per individual (6 measurements for the first set and 2 measurements for the second set). Figure S7 shows the the pairwise posterior correlation plot of the model parameters, based on the two datasets. Parameters are well identified in both cases but the uncertainty in the parameter posterior is higher for the case with only eight measurements.

	mean	true	sd	n_{eff}	Rhat
β	-9.94	-10	0.06	2111	1.00
μ_U	199.80	200	0.20	232	1.02
σ_U	1.10	1	0.09	113	1.05
σ_{HI}	4.01	4	0.06	3492	1.00
σ_{EIA}	0.51	0.5	0.01	3933	1.00
S_{EIA}	14.76	15	0.11	2181	1.00

Table S2: Posterior estimates of the population-level parameters when 50 measurements were used. The HMC sample size was 4000.

	mean	true	sd	n_{eff}	Rhat
β	-9.77	-10	0.16	5116	1
μ_U	199.64	200	0.32	3915	1
σ_U	1.02	1	0.09	1127	1
σ_{HI}	3.90	4	0.13	7548	1
σ_{EIA}	0.58	0.5	0.05	1864	1
S_{EIA}	14.65	15	0.30	5050	1

Table S3: Posterior estimates of the population-level parameters when 8 measurements were used. The HMC sample size was 4000.

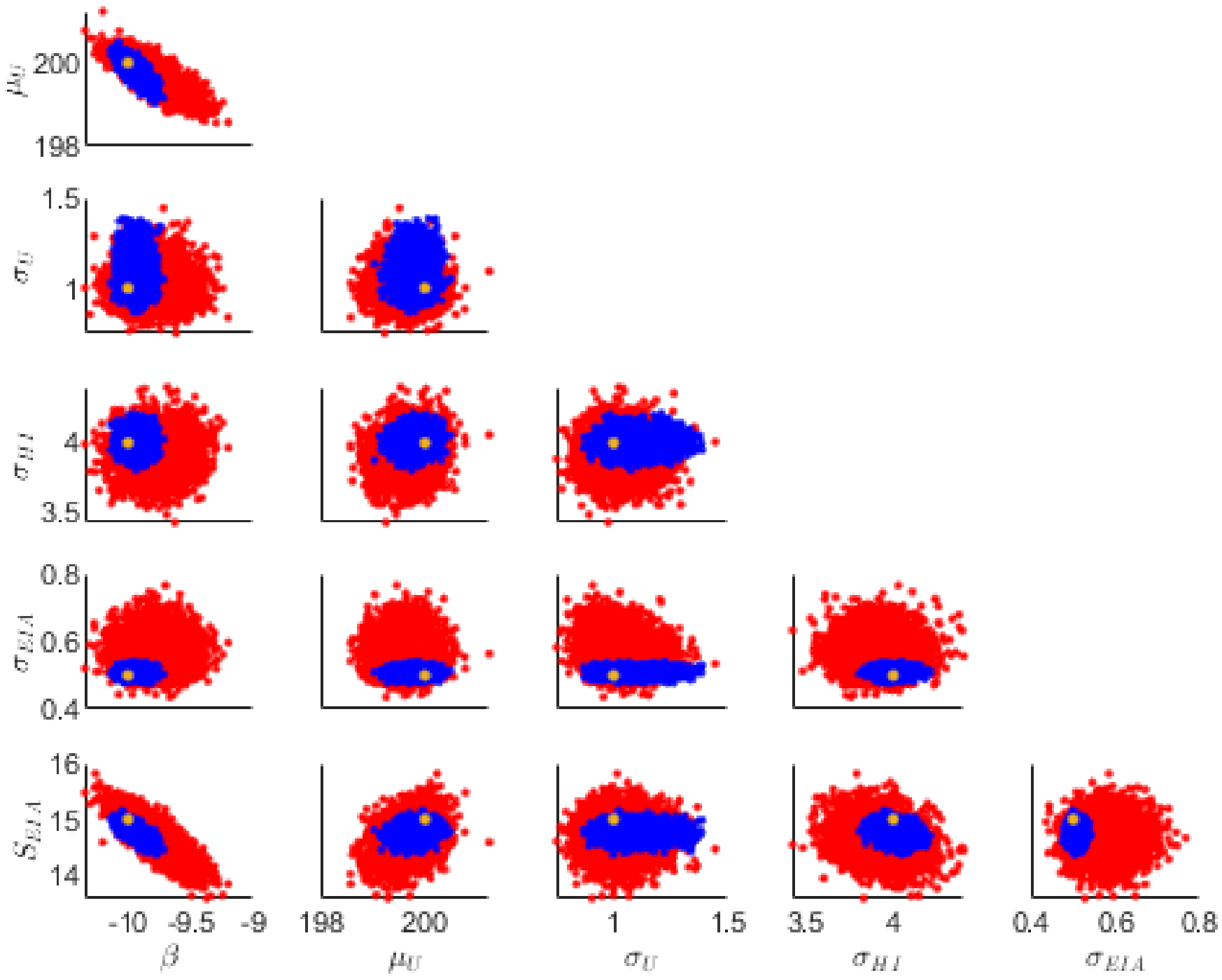

Figure S7: Correlation plot for the parameters of the two sets of measurement, estimated independently: blue is 50 measurements, red is for 8 measurements and the Yellow dot denote the true values.
